# Differential inter-subject correlation of brain activity when kinship is a variable in moral dilemma

**DOI:** 10.1038/s41598-017-14323-x

**Published:** 2017-10-27

**Authors:** Mareike Bacha-Trams, Enrico Glerean, Robin Dunbar, Juha M. Lahnakoski, Elisa Ryyppö, Mikko Sams, Iiro P. Jääskeläinen

**Affiliations:** 1 Brain and Mind Laboratory, Department of Neuroscience and Biomedical Engineering, Aalto University, Espoo, Finland; 2 Turku Pet Centre, University of Turku, Turku, Finland; 3 Social and Evolutionary Neuroscience Research Group, University of Oxford, Oxford, United Kingdom; 4 Department of Computer Science, Aalto University, Espoo, Finland; 5 Independent Max Planck Research Group for Social Neuroscience, Max Planck Institute of Psychiatry, Munich, Germany; 6 Advanced Magnetic Imaging (AMI) Centre, Aalto NeuroImaging, Aalto University, Espoo, Finland

## Abstract

Previous behavioural studies have shown that humans act more altruistically towards kin. Whether and how knowledge of genetic relatedness translates into differential neurocognitive evaluation of observed social interactions has remained an open question. Here, we investigated how the human brain is engaged when viewing a moral dilemma between genetic vs. non-genetic sisters. During functional magnetic resonance imaging, a movie was shown, depicting refusal of organ donation between two sisters, with subjects guided to believe the sisters were related either genetically or by adoption. Although 90% of the subjects self-reported that genetic relationship was not relevant, their brain activity told a different story. Comparing correlations of brain activity across all subject pairs between the two viewing conditions, we found significantly stronger inter-subject correlations in insula, cingulate, medial and lateral prefrontal, superior temporal, and superior parietal cortices, when the subjects believed that the sisters were genetically related. Cognitive functions previously associated with these areas include moral and emotional conflict regulation, decision making, and mentalizing, suggesting more similar engagement of such functions when observing refusal of altruism from a genetic sister. Our results show that mere knowledge of a genetic relationship between interacting persons robustly modulates social cognition of the perceiver.

## Introduction

Evaluating and predicting social interactions of others is an integral part of social cognition, one of the most fundamental of human cognitive functions. Indeed, the evolution of social cognition may best explain why humans have a more developed neocortex than other species^1^. So far, social cognition has been predominantly studied with stimuli depicting interactions between strangers, however, most of the significant interactions evaluated in daily life are between one’s family members, friends, and acquaintances^2,3^_._

More importantly, most of our social interactions (and social effort) is directed to a very small number of familiar individuals, 60% of our social effort is directed to just 15 close friends and family^4^. There is also considerable experimental and observational evidence for a “kinship premium” in our interactions with others, especially when those interactions involve altruistic behaviour^5–7^. We are more likely to help our genetic relatives compared to unrelated individuals, and to do so implicitly, without conscious elaboration^8,9^. In a trolley dilemma^10^, subjects have to decide if they would push a handle to set a trolley to another track so that instead of killing five people when left without intervention, it will kill a single person on an alternative track. If only strangers are considered, the study subjects favoured the survival of the five over one life; however, their judgement changed if the single person was genetically related to the subject.

On the other hand it has been shown, that subjects judged incest to be equally morally wrong for a sibling, irrespective of whether this was a genetic, adoptive or step sibling^11,12^. In these studies, rather co-residence with the sibling in the family, irrespective of genetic status, was the most relevant factor in decisions about the moral reprehensibility of incest.

These and many other studies, have shown differences in multiple aspects of moral perception/processing, evaluation, judgement and decision making at the behavioural level when processing information about kin vs. non-kin. However, much less is known about the neuronal underpinnings of these effects. Recently, Wlodarski and Dunbar^13^ have shown that different brain regions are active when subjects judge moral dilemmas about kin vs. friends. They found the sensorimotor cortex, ventromedial prefrontal cortex and posterior cingulate cortex to be more strongly activated when the subjects processed social information about their friends than about their kin. These differences imply that the brain processes kinship information differently than information on unrelated individuals.

We explored this further by comparing subjects’ brain responses to a moral dilemma involving a pair of genetic versus adoptive (*i*.*e*., unrelated) sisters. During functional magnetic resonance imaging (fMRI) the subjects viewed the same movie involving two sisters, but one group was primed with the information that they were genetic sisters and the other group with the information that they were sisters by adoption. The case of sisters related genetically vs. by adoption is especially suitable for testing whether knowledge of genetic relationship influences perception of a moral dilemma between kin given that there is no potential for shared genetic interest in future generations for adopted siblings^14^. Note, however, that the current study examined perception of a kin relationship that subjects were seeing in a movie, while in Wlodarski and Dunbars^13^ analysis the subjects answered questions about their own kin members and friends.

In the present study, we utilized inter-subject correlation (ISC) of brain hemodynamic activity as a model-free analysis approach that makes it possible to use movies as ecologically valid stimuli during fMRI. Due to improvements in fMRI acquisition methods and data analysis algorithms^15^, it has become possible to study specific aspects of social cognition between subjects using ecologically valid fMRI paradigms. The ecological validity is particularly important when studying moral dilemmas in order to engage the subjects and make the dilemma as credible and perceptible as possible in order to get authentic reactions. To investigate the degree of similarity in how individual brains respond to the common movie stimulus, the brains of individual subjects are aligned and ISC between the hemodynamic activity time courses for each voxel are calculated across all subject pairs. ISC can be interpreted as reflecting synchronized neural activity and thus similarity of cerebral information processing across individuals^15–18^. It has been shown that when viewing a feature film during brain scanning, both “higher-order” prefrontal cortical as well as basic sensory cortex regions become synchronized across subjects^19,20^. Further, ISC may not only reflect mutual neuronal responses, but could provide the basis of inducing a specific common mind set, e.g. built by context information or perspective taking as well as predicting the actions of others^21,22^.

The model-free approach of ISC does not require any a priori, pre-designed modeling of the fMRI signal to carry out the analysis and thus provides a powerful tool to investigate neuronal mechanisms as the correlations are exclusively based on similarities between the subjects’ brain activities when they react to the various aspects of the complex movie^23^. At the same time, ISC has been shown to reliably detect involved brain regions in complex experimental setups almost as sensitively as a model-based analysis^24^.

In study 1, we asked whether people discriminate behaviourally between relatives with genetic vs. non-genetic backgrounds: In an implicit association test (IAT^25^) the subjects’ reaction time when associating the words “sister” and “adopted sister” to positive or negative connoted adjectives was measured. Further, after watching the movie, the subjects were asked whether genetic vs. non-genetic relationship status mattered to them in the moral dilemma that they observed.

In study 2, we tested how the subjects perceived moral dilemmas involving genetically related vs. unrelated individuals during fMRI. In a first task, the subjects watched the movie depicting the moral dilemma between two sisters after being primed that they were either genetically related sisters or sisters related only by adoption. Should knowledge of genetic relationship matter, we expect to see differences in the behavioural tests (IAT; questionnaires) as well as the neuronal mechanisms: we predict that brain regions known to be involved in processing of mentalizing^26,27^, conflict resolution^28,29^, emotion regulation^30,31^, and moral dilemmas^32,33^ would be activated differently under the two viewing conditions.

Second, following the hypothesis that moral processing is the most relevant factor to distinguish between watching the movie when believing the sisters to be genetic or adopted (i.e., non-genetic), each subject underwent a moral decision task during fMRI scanning to evaluate specifically which brain areas are associated with the perception and processing of moral dilemmas during movie-watching. In this task the subjects had to decide whom to save from a dangerous area and had different choices including their own sister, best friend, and strangers, an experimental design similar to the classical moral trolley dilemma. Again, if the genetic relationship had an effect on the viewers, as has previously been shown behaviourally^10^, we hypothesize that the subjects will show kinship preference by saving their sister over others and that similar brain areas are engaged both in the decision task and when watching the movie believing that the sisters are genetic.

## Results

### Study 1: Implicit association test (IAT)

To examine if a possible general implicit bias against adopted sisters (that potentially modulates brain functions during movie viewing) underlies the subjects’ perception of the movie, we asked 30 subjects in a behavioural experiment to undergo an IAT^25^. In this test, reaction times during assignment of positive and negative connoted words to the categories of sister and adopted sister showed that there is no such implicit bias: Out of 30 subjects, nine favoured a genetic sister, 13 a non-genetic sister, and eight had no preference (one sampled t-test t = −0.9564 p = 0.3468). The TOST procedure^34^ indicated that the ratings of emotional closeness were significantly similar (observed effect size (d = −0.17) was significantly within the equivalent bounds of d = −0.68 and d = 0.68; t(29) = 2.77, p = 0.005).

### Study 2: Inter-subject correlation (ISC) of fMRI during movie watching

#### Inter-subject correlation (ISC) across all conditions

**Figure 1:**
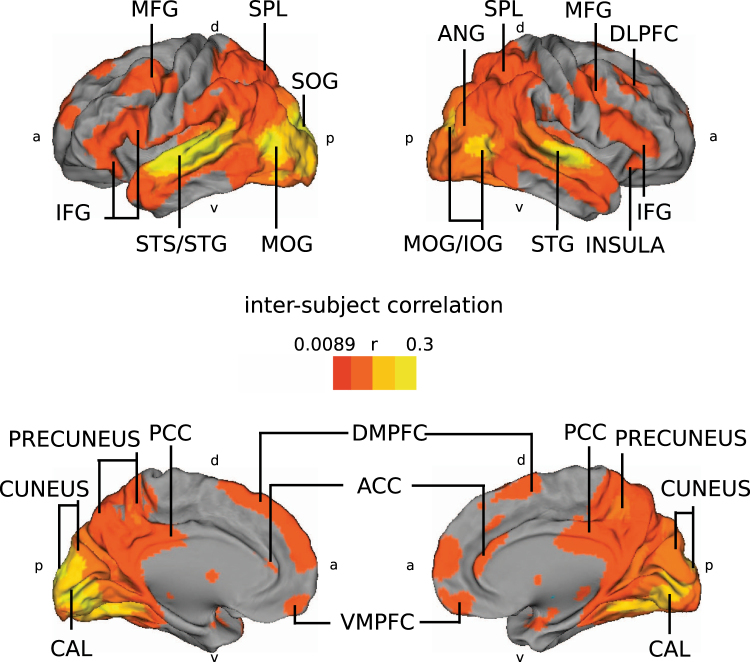
Inter-subject correlation (ISC) of all 30 subjects during the first viewing of the movie. On top row are shown lateral and on bottom row medial surfaces of left and right cerebral hemispheres. Red-yellow colours indicate areas of significant ISC during movie watching (FDR q < 0.05). Abbreviations: ACC = anterior cingulate cortex, ANG = angular gyrus, CAL = calcarine gyrus, DLPFC = dorsolateral prefrontal cortex, DMPFC = dorsomedial prefrontal cortex, IFG = inferior frontal gyrus, IOG = inferior occipital gyrus, MFG = middle frontal gyrus, MOG = middle occipital gyrus, MTG = middle temporal gyrus, PCC = posterior cingulate cortex, SOG = superior occipital gyrus, SPL = superior parietal lobe, STS/STG = superior temporal sulcus and gyrus, VMPFC = ventromedial prefrontal cortex, a = anterior, d = dorsal, p = posterior, v = ventral.

During fMRI scanning the subjects watched a movie depicting a moral dilemma between two sisters, either believing the sisters are genetic sisters or that the younger sister was adopted at birth. In a first step, the overall ISC (22) of hemodynamic activity of an independent set of 30 subjects was calculated during first viewing of the movie (Fig. 1). Significant ISC was observed extensively in occipital lobes, posterior parietal areas, and temporal cortices. In the frontal cortex, areas in the lateral inferior frontal gyrus (IFG), lateral middle frontal gyrus (MFG), dorsolateral prefrontal cortex (DLPFC), dorsomedial prefrontal cortex (DMPFC) and ventromedial prefrontal cortex (VMPFC) showed ISC between all subjects. The location of all brain areas were defined using anatomical brain atlases as specifically the Harvard-Oxford Cortical Structural Atlas and the Juelich Histological Atlas.

#### Differences in ISC between conditions

**Figure 2:**
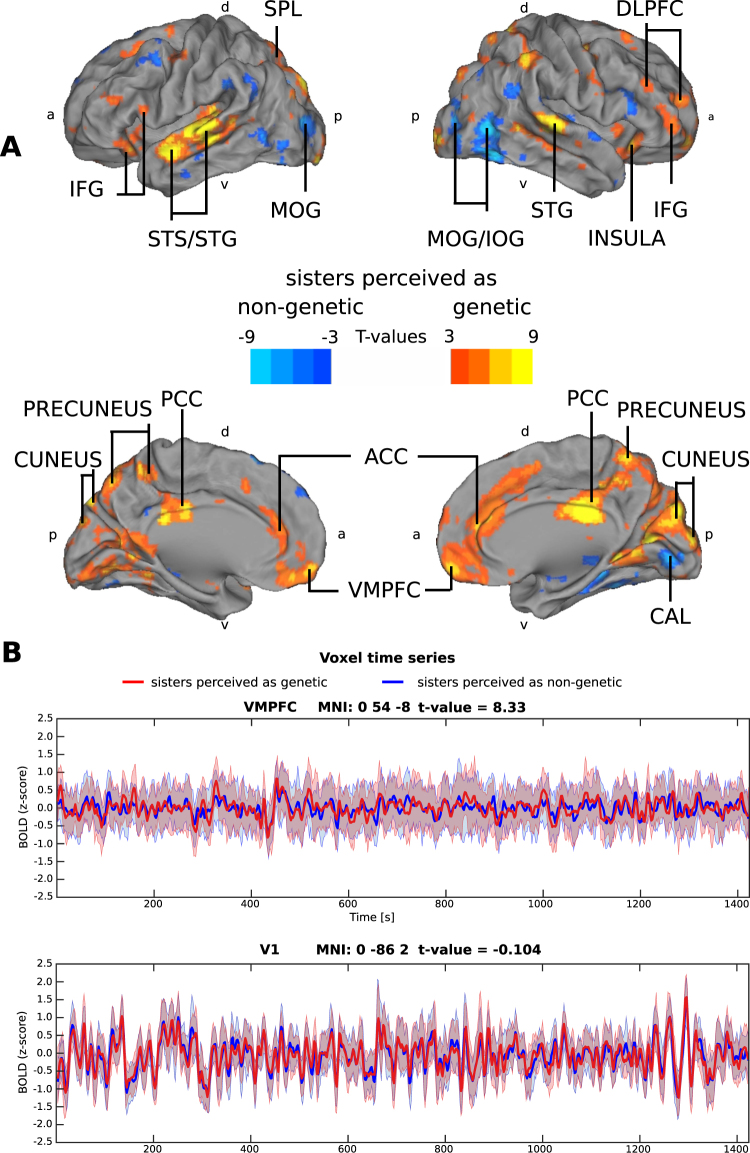
Differential ISC between the conditions of an assumed genetic and non-genetic sisters and BOLD time series from two exemplary single voxels. (**A**) Significant differences in brain activity when all subjects watched the movie thinking that the sisters were genetically vs. non-genetically related (FDR q < 0.05, t = 2.1447, for consistent illustration purposes, the figures shows t-values from 3 to 9 and −3 to −9) (N = 30, within subject design). Red-yellow colours indicate areas of significantly higher ISC when the subjects watched the movie as depicting genetically related, as compared to non-genetically related, sisters. Blue colour indicates areas showing significantly higher ISC in the reverse contrast. Abbreviations: ACC = anterior cingulate cortex, CAL = calcarine gyrus, DLPFC = dorsolateral prefrontal cortex, IFG = inferior frontal gyrus, IOG = inferior occipital gyrus, MOG = middle occipital gyrus, PCC = posterior cingulate cortex, SPL = superior parietal lobe, STS/STG = superior temporal sulcus and gyrus, VMPFC = ventromedial prefrontal cortex, a = anterior, d = dorsal, p = posterior, v = ventral. (**B**) Across subjects averaged BOLD time series of two voxels, one in the area VMPFC that showed significantly higher ISC when the subjects were viewing the sisters as genetic and one time series of a voxel in area V1 (primary visual cortex) that did not show significant between-condition ISC differences. The red line plots the group mean BOLD in the genetic sisters condition and the blue line plots the group mean BOLD in non-genetic sisters condition over the whole length of the movie. Red and blue shades indicate the 25^th^ and 75^th^ percentile of the variance.

In a second step, the ISC of all subjects (N = 30) were contrasted between the genetic vs. non-genetic relationship viewing conditions. As each participant watched the movie in the genetic and in the non-genetic condition on two different scanning days in a counterbalanced order, this is a within-subject design. There were robust differences between the two conditions in the ISC of hemodynamic activity of the subjects, despite 90% of the subjects self-reporting that it did not matter to them whether the sisters were related genetically or not. When the subjects watched the movie believing that they were seeing genetically related sisters, the ISC was significantly stronger in the superior temporal sulcus and gyrus (STS/STG), VMPFC, DLPFC, anterior cingulate cortex (ACC) and posterior cingulate cortex (PCC), IFG, insula, cuneus, precuneus, and superior parietal lobule (SPL) (Fig. 2, Table 1).

**Table 1:** Clusters size, peak coordinates and t value of all clusters of Experiment 1 (movie watching task).

Cluster Label: movie watching task	Cluster extent (voxels)	x MNI (mm)	y MNI (mm)	z MNI (mm)	Peak value (T-value)
**clusters genetic > non-genetic**					
Superior parietal lobule (R)	8794	8	−86	44	14.6272
Ventromedial prefrontal cortex (R)	4099	0	54	−8	8.3312
Superior temporal gyrus (L)	2182	−42	−26	6	16.974
Superior temporal gyrus (R)	1138	62	−26	4	13.0493
Putamen (R)	616	18	16	−10	7.2211
Insula (L)	266	−40	18	−10	6.9642
Inferior temporal gyrus (R)	145	52	−46	−30	7.432
Middle temporal gyrus (R)	110	66	−4	−16	10.4489
Inferior frontal gyrus (L)	89	−36	34	20	5.3232
Superior frontal gyrus (R)	89	8	62	26	6.2544
Inferior parietal lobule (L)	86	−52	−60	48	5.0215
Postcentral gyrus (R)	81	56	−6	38	7.0252
Superior parietal lobule (L)	73	−14	−58	72	7.4174
**clusters non-genetic > genetic**					
Inferior occipital gyrus (R)	1938	40	−66	−8	−12.3679
Middle occipital gyrus (L)	604	−32	−84	4	−9.7755
Cerebellar crus II (R)	318	30	−86	−32	−7.3011
Temporal pole (middle) (R)	171	38	22	−34	−6.6407
Inferior temporal gyrus (L)	165	−54	−64	−4	−8.2858
Angular gyrus (L)	147	−42	−54	28	−5.5752
Superior temporal gyrus (R)	95	64	−28	20	−8.3381
Inferior frontal gyrus (R)	94	52	20	16	−5.7279
Superior temporal gyrus (R)	87	64	0	−4	−9.6944
Precentral gyrus (L)	86	−44	10	52	−6.1405
Superior frontal gyrus (L)	86	−8	20	60	−7.8522

When the subjects thought that the sisters were non-genetic, higher ISC was observed mainly in the occipital cortex. Importantly, the movie stimulus was identical in both viewing conditions.

To illustrate blood oxygenation level dependent (BOLD) time series of specific voxels, in Fig. 2, the panel B shows the time series of two exemplary voxels, over the whole length of the movie, from: i) VMPFC that showed higher ISC when the subjects viewed the sisters as genetic and ii) from a voxel in the area V1 (primary visual cortex), which is an early sensory brain area that did not show any between condition differences in ISC.

**Figure 3:**
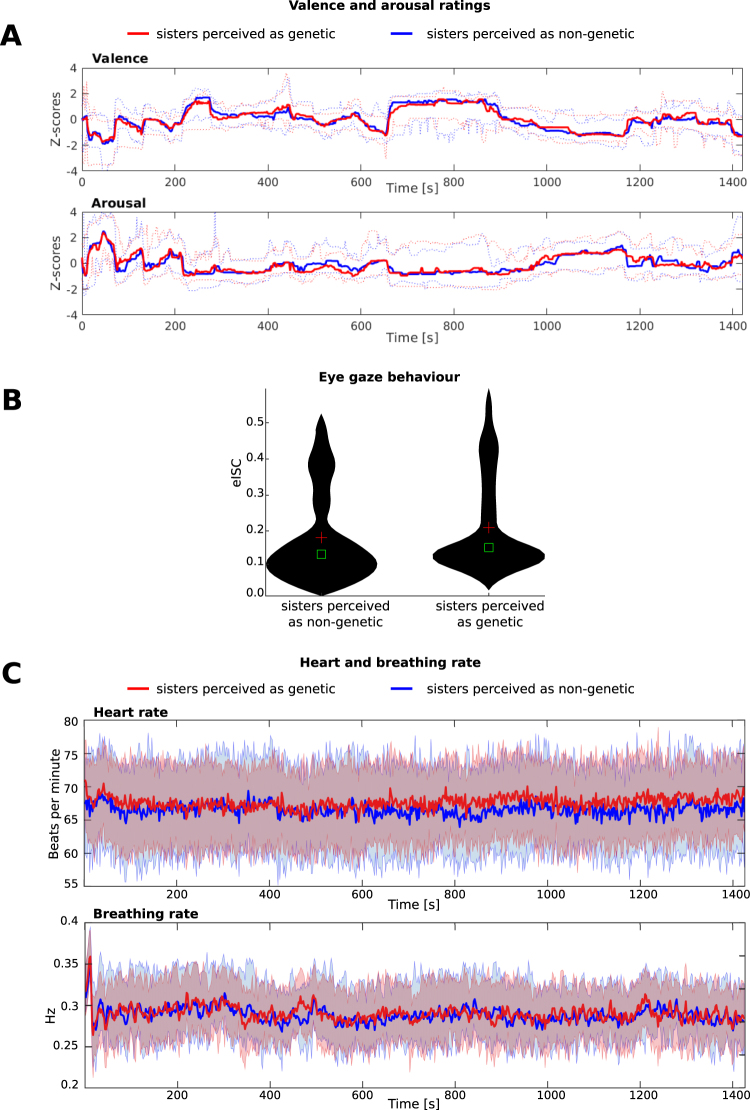
Experienced emotional valence and arousal as well as physiological parameters when perceiving the sisters in the movie as genetic vs. non-genetic. (**A**) Shown are dynamic self-ratings of emotional valence and arousal over the whole time course of the movie obtained during re-viewing of the movie after the fMRI sessions when the sisters were viewed as genetically related (red) or non-genetic (blue). The ratings were highly similar and there were no time periods where significant between-condition differences could have been observed. Note that half of the subjects (N = 2 × 15) rated experienced arousal and the rest rated experienced valence after the first fMRI session followed by rating the other emotional dimension after the second fMRI session. Plotted are means for all subjects in the red line for assumed genetic sisters and the blue line for non-genetic sisters condition. Red and blue dashed lines show the 25^th^ and 75^th^ percentile of the variance. (**B**) Eye gaze behavior (N = 29) in the movies when the sisters were perceived as either genetic (right) or non-genetic (left) shown as a violin plot with the red cross depicting the means and green squares the medians. There were no significant differences between the conditions. (**C**) Breathing and heart rates (N = 30) when the sisters were perceived as either genetic (red) or non-genetic (blue). There were no significant differences between the conditions. Red line plots the condition with assumed genetic sisters and the blue line non-genetic sisters. Red and blue shade show the 25^th^ and 75^th^ percentile of the variance.

Self-ratings of emotional valence and arousal obtained after the scans were not significantly different between the conditions (Valence: r = 0.0075, p = 0.3458, Arousal: r = −0.0189, p = 0.6081) (Fig. 3). Further, the mean ISC of eye-movements (eISC) over time windows showed no significant difference between the groups of participants believing in genetic or non-genetic sisters (p = 0.3918) (see Fig. 3). Likewise, no significant difference could be found in the heart and breathing rate comparing the conditions of assumed genetic versus non-genetic sisters (with bootstrap over 5000 permutations, breathing rate: t-value = 0.430, p = 0.335: heart rate: t-value = −1.12, p = 0.129) (see Fig. 3).

### Comparison with the moral dilemma decision experiment

**Figure 4:**
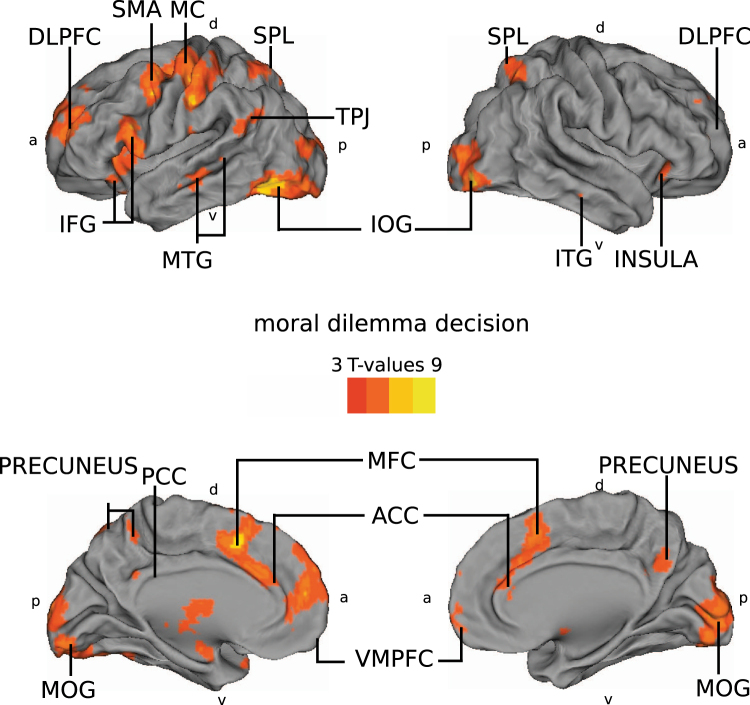
Having to decide in a simulation between saving one’s sister, friend, and others from crisis regions elicited significant brain activity in the VMPFC, ACC, precuneus, DLPFC, IFG, insula, TPJ and MTG. These activations of subjects (N = 30) were obtained by contrasting the decision phases (from the point of revealing the individuals involved until the decision signaled by the subject’s button press) against non-decision phases (subjects watching the background story depicting the two crisis regions and how the subjects only have resources to save individuals from one of the crisis regions) (FDR q < 0.05, t = 2.0384, for consistent illustration purposes, the figures shows t-values from 3 to 9) Left-lateralized motor and supplementary motor are probably explained by the button press that the subjects performed to announce their decision. Abbreviations: ACC = anterior cingulate cortex, DLPFC = dorsolateral prefrontal cortex, IFG = inferior frontal gyrus, IOG = inferior occipital gyrus, ITG = inferior temporal gyrus, MC = motor cortex, MFC = medial frontal cortex, MOG = middle occipital gyrus, MTG = middle temporal gyrus, PCC = posterior cingulate cortex. SMA = supplementary motor area, SPL = superior parietal lobe, TPJ = temporo-parietal junction,VMPFC = ventromedial prefrontal cortex, a = anterior, d = dorsal, p = posterior, v = ventral.

To further examine which neurocognitive processes might be involved, we studied whether the brain areas showing higher ISC in the genetic condition overlap with those engaged during a modified moral dilemma task^35^ analysed with general linear modelling (GLM) (Fig. 4, Tables 1 and 2). Naturally, it should be kept in mind that while ISC and GLM analyses of brain hemodynamic activity can yield converging results^24^, this is not necessarily the case, as high ISC can be observed also when the BOLD signals are small.

**Table 2:** Clusters size, peak coordinates and t value of all clusters of and Experiment 2 (moral dilemma decision task).

Cluster Label: moral dilemma decision task	Cluster extent (voxels)	x MNI (mm)	y MNI (mm)	z MNI (mm)	Peak value (T-value)
Cerebellar lobule VI (R)	10417	20	−54	−30	10.1921
Superior frontal gyrus (L)	3631	−8	10	48	8.4473
Precentral gyrus (L)	2476	−34	−18	58	7.2107
Inferior frontal gyrus (L)	1207	−52	12	22	6.3788
Superior parietal lobule (L)	815	−28	−56	48	6.0945
Amygdala (L)	720	−14	−12	−12	6.2453
Putamen (R)	257	24	10	8	5.4598
Superior parietal lobule (R)	199	26	−62	54	4.8181
Angular gyrus (L)	156	−48	−52	22	4.9727
Insula (R)	128	40	22	0	5.3664
Middle temporal gyrus (L)	123	−62	−24	−12	4.7632
Temporal pole (middle) (L)	77	−56	8	−24	5.4375

**Figure 5:**
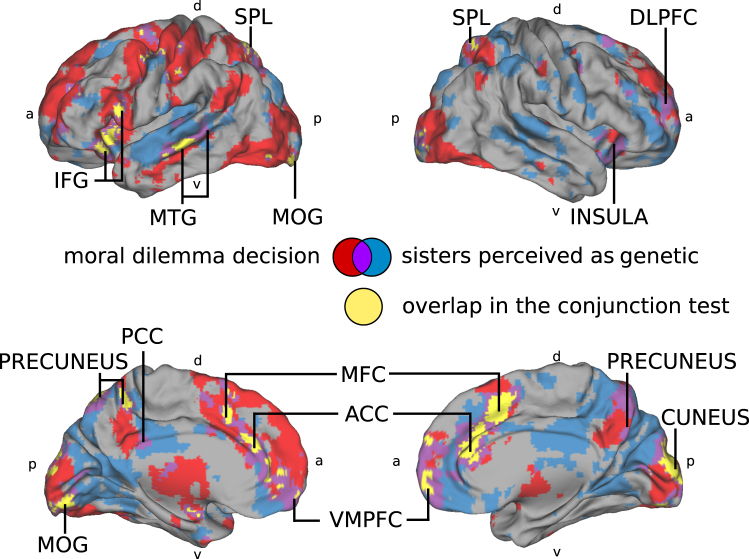
Activity during moral dilemma decision making as disclosed by GLM analysis (red) and the ISC when the subjects believed in a genetic relationship between the sisters in the movie (blue), along with the overlap of these two maps (violet), as well as the more strict overlap with a conjunction test (yellow); ^64^, (FDR q < 0.05, t = 2.0384 for GLM and t = 2.1447 for ISC). Abbreviations: ACC = anterior cingulate cortex, DLPFC = dorsolateral prefrontal cortex, IFG = inferior frontal gyrus, MFC = medial frontal cortex, MOG = middle occipital gyrus, MTG = midddle temporal gyrus, PCC = posterior cingulate cortex. SPL = superior parietal lobe, VMPFC = ventromedial prefrontal cortex, a = anterior, d = dorsal, p = posterior, v = ventral.

When the same subjects who watched the movie had to decide between saving their sister, best friend, vs. stranger(s), in various combinations, from a crisis region, 93% of the subjects showed a clear kin preference by choosing their sister (even when associated with some strangers) rather than their best female friend (chi-squared test ×^2^ = 43.4 p < 4 × 10^−11^). Further, as can be seen in Fig. 5 the VMPFC, ACC, IFG, MTG, SPL, PCC, precuneus, DLPFC, and anterior insula were consistently involved in both making choices of whom to prefer in a morally dilemmatic situation and when observing the moral dilemma between a genetic vs. non-genetic sister. Importantly, ratings of emotional closeness were not significantly different for the subjects’ sisters and their best friends with an average of 9.28 (sisters) and 8.80 (friends) on a 1–10 scale (Wilcoxon signed rank test = 0.12, t-test, t = 1.64 p = 0.11). The TOST procedure^34^ indicated that the ratings of emotional closeness were significantly similar (observed effect size (d = 0.33) was significantly within the equivalent bounds of d = −0.68 and d = 0.68, or in raw scores: −1.07 and 1.07, t(29) = −1.92, p = 0.032).

As measured with an independent group of subjects outside the scanner, reaction times for the moral-dilemma decision were significantly longer in the case of a decision between a group comprised of their friend and four strangers on one side and their sister alone on the other side as in the case that only comprised strangers (on both sides) (paired Wilcoxon rank sum test p = 0.0011973).

## Discussion

In the present study, we investigated whether refusing altruism from a sister is perceived differently when the viewers think that the sisters are genetically related vs. when they think that one of the sisters has been adopted at young age. The results of the IAT in study 1 suggest that the subjects do not show an implicit bias against adoptive sisters compared to genetic sisters in general. Also, when explicitly asked if the relationship (as genetic or adopted) would matter in the decision of an organ donation, most subjects (90%) report that this knowledge would not affect the decision. Further, heart rate and breathing rate exhibited no significant differences between the two conditions, and self-reported emotional valence and arousal was likewise similar between the conditions (Fig. 3), suggesting that there were no robust differences in experienced emotions between the conditions.

In contrast, the ISC of the hemodynamic brain activity show a different picture: robust differences were observed in patterns of brain activity due to the mere knowledge of the genetic relationship between the sisters in the movie. Specifically, there were multiple brain regions showing significantly higher ISC when the subjects thought that they are seeing a young girl refusing to donate her organ to save her genetic, as opposed to non-genetic, sister (Fig. 2). These areas included VMPFC, DLPFC, ACC, PCC, insula, precuneus, and SPL. While we would caution against drawing conclusions of specific cognitive functions involved on the basis of observed differences in brain activity, these brain regions have been previously shown to be associated with moral and emotional conflict regulation^36^, decision making^37^, mentalizing^32^, and perspective taking^38^, thus tentatively suggesting more uniform engagement of such neurocognitive functions when observing the dilemma of organ donation between genetic sisters.

In the reverse contrast (i.e. when the subjects thought the sisters were non-genetic), higher ISC was observed in brain areas in the occipital cortex, conventionally associated mainly with visual perception^39,40^. One possible explanation could potentially be that in the case of a non-genetic relationship between the sisters, the processing of complex social conflict associated with the moral dilemma is less demanding, and therefore leaves room for the subjects to focus on the visual aspects of the movie. However, eye-movements were not significantly different between the two viewing conditions, suggesting that differences in attention to movie events did not explain the observed robust differences in ISC.

To specifically test for the possibility that the differences in ISC between the conditions reflected differences in moral evaluation, we compared the areas showing differences in ISC with areas activated when the subjects engaged in a separate moral-decision making control task. In this control task, the subjects had to make choices about saving people (including their sister, best friend, and strangers, in various combinations) from disaster. As each subject made only one decision that contrasted saving the friend over strangers and one decision of saving the friend over the own sister plus four strangers (as well as four decisions that contrasts the sister to groups of others), the statistical power in this experimental design was unfortunately not sufficient to differentiate directly between brain responses during decisions to save the sister vs. the friend. Rather, the results should be viewed as localization of brain regions involved in making moral decisions, yet also modulated by differences in, e.g., executive control, readiness for action, and attention, between the passive perception of the story and active decision making. However, significantly longer reaction times suggested increased difficulty when having to choose between a sister and the friend together with four strangers while areas in the VMPFC, DLPFC, ACC, PCC, precuneus, IFG, MTG, SPL and anterior insula were consistently involved in both making choices of whom to prefer in a morally dilemmatic situation and when observing the moral dilemma between a genetic vs. non-genetic sister (Fig. 5). This overlap in engaged brain regions suggests that processing of moral dilemmas took place during movie watching when the sisters were understood to be genetically related. It is significant that the brain regions flagged up in this analysis are those known to be involved in processing moral dilemmas and mentalizing. The DLPFC has been reported to play a role in overcoming a primary moral judgment in favour of greater welfare^26,41^ and in cortical emotional processing^42^, while the MTG has been implicated in attributing mental states as well as ingroup/outgroup distinctions^33,43,44^, the ACC has been reported to be engaged in resolving conflicts^28,29^, and the SPL, precuneus, and PCC have been implicated in mentalizing and perspective taking^27,45,46^. Further, the VMPFC has been associated with viewing moral conflicts, making moral decisions, attributing mental states to self and others, adopting another person’s perspective, and evaluating their beliefs^31,38,46,47^.

Thus, the differences in ISC between the conditions appear to have arisen due to the knowledge about the sisters’ relationship influencing cognitive evaluation of the moral dilemma depicted in the movie. The contrast to the behavioural results assessed in study 1, where we find that behavioural decisions were not influenced by knowledge of the relationship, is particularly interesting since it suggests that differential processing is taking place under the surface.

There could be at least two possible explanations for these findings. First, the study subjects might have purposely been hiding their “real” honest opinions as they might have not been willing to reveal these to the researchers, presumably because of social pressure against discriminating between genetic and adoptive siblings. However, the IAT is an implicit test for biases (it uses differences in reaction times for associations of a specific term with positively and negatively connoted words), so subjects are not aware of their performance on this task. Further, they do not know the exact measures which are used to calculate the IAT score, thus making it difficult to engineer potential biases; hence, it is very difficult, even impossible, to manipulate an IAT response in a desired direction^48^. Thus, while a conscious manipulation of reported opinions would be possible in the open-format questionnaire (when asked if the relationship of the sisters matters in the situation of organ donation), it is very unlikely in the IAT. However, Liberman *et al*.^11^ showed that, when judging incest reprehensibility, the coresidence of siblings is a stronger factor than the assumed relationship status and in when the two parameters are in conflict, the time spent in coresidence outweighs the belief of kin relation. As in this study the subjects were told that the apparent adoption took place as the younger sister was a newborn (implying coresidence of the sisters in both the genetic and the adoption case), we suggest, in accordance with Liberman *et al*., that the factor of coresidence was given greater account than the kin relationship and thus the subjects’ explicit answers in the questionnaire could probably be seen/taken as truthful i.e. reporting authentic, honest thoughts.

A second possible explanation for our findings is that, as the results of the implicit testing show, the study subjects indeed did not show any biases behaviourally and still pursued different ways of considering the case of a relationship by genes or by adoption, with resulting differences in brain activity patterns. These results show that an event that is behaviourally counter-intuitive (e.g. refusing to help a sister to prolong her life) needs different and potentially more intensive mental processing when the sisters are related genetically compared to adoptive sisters. As the differences in the brain activity patterns between the genetic and the adopted condition particularly comprise areas known to be involved in processing moral dilemmas and mentalizing as the VMPFC, DLPFC, ACC, PCC, precuneus, IFG, MTG, SPL, and anterior insula, we suggest that the study subjects’ expectations of morality are more strongly violated when close genetic relatives refuse to help each other than when unrelated individuals behave this way, despite their close social relation (adoption).

Notwithstanding these points, we wish to caution the reader to keep in mind the caveats associated with reverse inference^49^ (although see^50^). Specifically, even as we are suggesting that an activation observed in a certain region is indicative of a specific cognitive process based on results of previous research documented in the literature, it is possible that the activation of that region in the present study was due to some other cognitive process. This is because in general any given brain region is involved in multiple cognitive functions, thus making it difficult to infer with certainty the cognitive functions involved in a task based on brain regions that are activated.

An alternative possibility is that the different measures operate at different levels of cognition: The null result in the IAT could be relying on a more basic level of attention to the social knowledge, whereas the questionnaire requires high level explicit cognition and the ISC during movie perception reflects some intermediate level of cognition. Had we thought to include them, other behavioural tests might have revealed more detail and background information on the subjects. Finally, it is always possible that something other than moral considerations could underlie the differences in brain patterns that we found, although, given the brain areas that show differences, this is unlikely.

In summary, we observed robust differences in brain activity when subjects viewed a movie depicting refusal to donate an organ to a genetic vs. non-genetic sister. These differences in brain activity were observed despite the subjects self-reporting that the relational status of the sisters did not make any difference to them. Areas of increased synchrony in the case of genetic sisters overlapped with those activated in a separate moral dilemma decision task. Taken together, our results suggest that the precuneus, MTG, insula, SPL, and the VMPFC, along with the associated cognitive processes (i.e., moral and emotional conflict regulation), decision making, mentalizing and perspective taking are synchronized across subjects more robustly when they are viewing refusal of altruism from genetic as opposed to non-genetic, kin. Overall, these findings are fundamentally important for understanding social cognition, a pivotal ability that makes us human and, among other things, enables the existence of societies. Our findings point out that the perceived relationship of interacting persons robustly modulates how the brains of spectators process third-party interactions. This is highly significant given that majority of research to date on social cognition has been on strangers, whereas most of our social interactions take place between family members, friends, and acquaintances.

## Material and Methods

### Subjects

We studied 33 healthy female subjects^51^ (19–39 years, mean age of 26 years, one left-handed, laterality index of right handed 84.5%). None of the subjects reported any history of neurological or psychiatric disorders. When asked, all subjects reported either normal vision or corrected to normal vision by contact lenses. Three subjects were excluded due to discomfort in the scanner, so that the final analysis included 30 subjects. 27 of them were native Finnish speakers and three were native Russian speakers. All subjects were sufficiently proficient in English to follow the dialogue in the movie without subtitles. The experimental protocols were approved by the research ethics committee of the Aalto University and the study was carried out with their permission (Lausunto 9 2013 Sosiaalisen kognition aivomekanismit, 8.10.2013) and in accordance with the guidelines of the declaration of Helsinki^51^. Written informed consent was obtained from each subject prior to participation.

### Stimuli and Procedure

**Figure 6:**
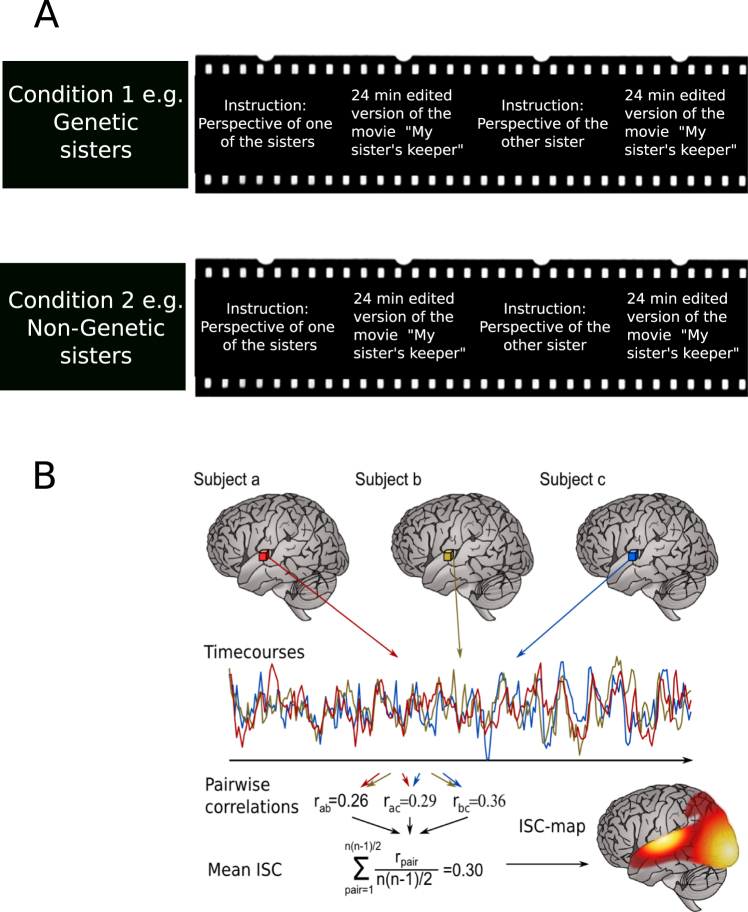
Experimental procedure and ISC analysis in the movie watching task. (**A**) Every subject watched the movie four times, in a 2 × 2 design assuming that the movie characters are either genetic sisters or not genetically related and taking the perspective of the to-be-donor sister Anna or the to-be-recipient sister Kate. The order of all the conditions were counter-balanced. (**B**) Time series from each voxel from all the fMRI recordings are compared across subjects in pairwise correlations to obtain the mean inter-subject-correlation (ISC).

The study consisted of two experiments. In the first experiment, the feature film *My Sister’s Keeper”* (dir. Nick Cassavetes, 2009, Curmudgeon Films), edited to 23 minutes and 44 s, (of which 14 min 17 s (60%) portray the theme of refusal of the organ donation),with the main story line retained, was shown to the subjects during fMRI. This shortened version of the movie focuses on the moral dilemma of the protagonist Anna to donate one of her kidneys to her sister Kate, who is fatally ill from cancer. In the course of the movie, Anna refuses to donate and Kate dies. The reason for Anna refusing to donate the kidney was not revealed to the subjects until after the experiment. The movie was shown to the subjects in the scanner four times in two separate scanning sessions on two different days. For each viewing of the movie the instructions were varied regarding the information about the sister’s relationship and the perspective to take in this viewing (Fig. 6). Each subject thus watched the movie assuming that the sisters were genetic sisters or that the younger sister Anna had been adopted as a newborn. In addition each subject was asked to take either the perspective of the potential donor (Anna) or the perspective of the potential recipient (Kate) on separate viewings (and both under the condition of a genetic or non-genetic relation background). The order of the different viewing conditions was counterbalanced between the subjects.

In the second experiment, each subject carried out a moral-dilemma decision task during fMRI in order to localize brain regions that are related to moral decision making. For this purpose, a modified version of the classical trolley dilemma^35,53,54^, was shown to the subjects. Each subject had to choose between rescuing different individuals, including unknown individuals, their sister and a best female friend. A presentation showing text and pictures told a story about civil unrest in a fictive distant country. This country had two parts: one part very dangerous and the other much less dangerous. Different people are in both parts of the country. Subjects were also told that as they were very rich and owned an airplane, they could go there and rescue some of the people. However, due to the circumstances in the country they had to decide which group of people to rescue. The two choices were always a group of five individuals on one side and a single person on the other. In seven runs the identity of the involved individual(s) was varied using the real names of the subject’s sister and best female friend. The 7 runs were: 1. All persons are unknown; 2. Sister is with four others in the dangerous part of the country, the single person is unknown; 3. Five persons are in the dangerous part of the country, the single person is the sister; 4. Five persons are in the dangerous part of the country, the single person is the friend; 5. Sister is with four others in the dangerous part of the country, the single person is the friend; 6. Friend is with four others in the dangerous part of the country, the single person is the sister; 7. Sister is with four others in the less dangerous part of the country, the single person is unknown. Responses in the moral dilemma decision task were recorded with a button press on a LUMItouch keypad (Photon Control Inc.8363, Canada). For the all questions, it was calculated with which percentage the sister was chosen over the friend and the stranger(s); statistical significance was tested with a Chi^2^ test.

### fMRI acquisition

Before each scan the subjects were informed about the scanning procedures and asked to avoid bodily movements during the scans. All stimuli were presented to the subject with the Presentation software (Neurobehavioral Systems Inc., Albany, CA, USA), synchronizing the onset of the stimuli with the beginning of the functional scans. The movie was back-projected on a semitransparent screen using a data projector (PT-DZ8700/DZ110X Series, Panasonic, Osaka, Japan). The subjects viewed the screen at 33–35 cm viewing distance *via* a mirror located above their eyes. The audio track of the movie was played to the subjects with a Sensimetrics S14 audio system (Sensimetrics Corporation Malden, USA). The intensity of the auditory stimulation was individually adjusted to be loud enough to be heard over the scanner noise. The brain-imaging data were acquired with a 3T Siemens MAGNETOM Skyra (Siemens Healthcare, Erlangen, Germany), at the Advanced Magnetic Imaging center, Aalto University, using a standard 20-channel receiving head-neck coil. Anatomical images were acquired using a T1-weighted MPRAGE pulse sequence (TR 2530 ms, TE 3.3 ms, TI 1100 ms, flip angle 7°, 256 × 256 matrix, 176 sagittal slices, 1-mm3 resolution). Whole-brain functional data were acquired with T2*-weighted EPI sequence sensitive to the BOLD contrast (TR 2000 ms, TE 30 ms, flip angle 90, 64 × 64 matrix, 35 axial slices, slice thickness 4 mm, 3 × 3 mm in plane resolution).

A total of 712 whole-brain EPI volumes were thus acquired for each movie viewing. The number of whole-brain EPI volumes for the moral dilemma decision task varied individually depending on the decision made by each subject (median 267 whole-brain EPI volumes). Heart pulse and respiration were monitored with the Biopac system (Biopac Systems Inc., Isla Vista, California, USA) during fMRI. Instantaneous values of heart rate and breathing rate were estimated with Drifter software package^55^ (http://becs.aalto.fi/en/research/bayes/drifter/).

### fMRI preprocessing

Standard fMRI preprocessing steps were applied using the FSL software (www.fmrib.ox.ac.uk) and custom MATLAB code (available at https://version.aalto.fi/gitlab/BML/bramila/). Briefly, EPI images were corrected for head motion using MCFLIRT.

Then they were coregistered to the Montreal Neurological Institute 152 2 mm template in a two-step registration procedure using FLIRT: from EPI to subject’s anatomical image after brain extraction (9 degrees of freedom) and from anatomical to standard template (12 degrees of freedom). Further, spatial smoothing was applied with a Gaussian kernel of 6 mm full width at half maximum. High pass temporal filter at a cut-off frequency of 0.01 Hz was used to remove scanner drift. To further control for motion and physiological artefacts, BOLD time series were cleaned using 24 motion-related regressors, signal from deep white matter, ventricles and cerebral spinal fluid locations (see^56^) for details, cerebral spinal fluid mask from SPM8 file csf.nii, white matter and ventricles masks from Harvard Oxford atlas included with FSL). As a measure of quality control we computed framewise displacement to quantify instantaneous head motion. Out of all the 120 runs (30 subjects, 4 sessions each), 97.5% of the runs (117 runs) had 90% of time points (640 volumes) with framewise displacement under the 0.5 mm threshold suggested in^57^. For the remaining three runs, the number of time points under 0.5 mm were 639 (89.7%), 633 (88.9%), 489 (68.7%), i.e. only one session had a considerable amount of head motion. While head motion is a concern in connectivity studies as it can increase spurious BOLD time series correlations that are affected by the same amount of instantaneous head motion, with across-brain time series correlation, head motion is expected to reduce the SNR. However, to make sure that head motion similarity did not explain any group difference, we computed the same permutation test for the ISC also for average framewise displacement by estimating the similarity of two subjects as the distance between their average framewise displacement value. We found that similarity in average head motion was not different between the two viewing conditions (t-value = 0.255; p = 0.398 obtained with 5000 permutations).

### Inter-subject correlation (ISC) analysis of brain activity during movie watching

To investigate how similar the brain activity was across subjects in the different experimental conditions, we performed inter-subject correlation (ISC) using the isc-toolbox (https://www.nitrc.org/projects/isc-toolbox/)^58^. For each voxel the toolbox computes a similarity matrix between subject pairs and within same subject in all conditions, with the conditions being: (i) shared assumption that the movie’s sisters are genetically related, (ii) shared assumption that the younger sister was adopted, (iii) shared perspective of the to-be-organ-donor, and (iv) shared perspective of the to-be-organ-recipient. The total size of the similarity matrix is then 120 × 120 (4 conditions × 30 subjects) with each subject having two viewings for the genetic and two viewings for the non-genetic condition. The comparison between the conditions of the sisters to be perceived as either genetic sisters or non-genetic sisters results thus in a total of 1740 pairs per condition, as the similarity of BOLD time series during the two viewings (in either the genetic or the non-genetic condition) of each subject is compared with the two respective viewings of the other N-1 subjects. As the order of subjects does not matter, the final number of pairs in same conditions will be 2*2*(N-1)*N/2 = 1740 with N = 30. Each value of the correlation matrix is a result of the correlation between the BOLD time series of the pair of subjects considered for the selected voxel. We computed differences between experimental conditions by first transforming the correlation values into z-scores with the Fisher Z transform and then computing t-values and corresponding p-values using a permutation based approach^59^.

The Fisher-Z transformed correlations of the two perspectives were pooled for either the genetic or the non-genetic sisterhood.

Correction for the multiple comparison was performed with Benjamini-Hochberg false discovery rate (BH-FDR) correction at a q < 0.05, corresponding to a t-value threshold of 2.133. For visualization purposes, all results were also cluster corrected by removing any significant cluster smaller than 4 × 4 × 4 voxels. Summary tables were generated with an increased t-value threshold of 3. For the conjunction or “intersection–union test”^64^ the p values of the ISC and GLM results are pulled together by considering the maximum p-value at each voxel. Then, multiple comparisons correction is performed with the Benjamini-Hochberg false discovery rate procedure with an FDR threshold equal to q < 0.05.

Unthresholded statistical parametric maps can be found in neurovault: http://neurovault.org/collections/WGSQZWPH/.

#### Perspective taking

In the movie-viewing experiment, in addition to having the subjects to watch the movie in the conditions of sisters related by birth or by adoption, we had altogether four runs, so that on two of the runs the subjects were asked to view the movie from the perspective of the sister who was expected to donate the organ, and on two of the runs from the perspective of the to-be-recipient sister. Thus, there was one run wherein the subjects viewed the movie from the perspective of the to-be- donor thinking that the sisters were genetic, one run wherein the subjects viewed the movie from the perspective of the to-be- donor thinking that the sisters were non-genetic, one run wherein the subjects viewed the movie from the perspective of the to-be- recipient thinking that the sisters were genetic, and one run wherein the subjects viewed the movie from the perspective of the to-be- recipient thinking that the sisters were non-genetic.

As the results of this task open up a completely other aspect of the experiment with various results to discuss, which go beyond the scope and the space limitation of this article, they will be reported separately elsewhere. These conditions are mentioned here for reasons of describing the experimental procedures thoroughly so that it would be possible for others to replicate the study should they wish to do so.

### General linear model analysis of the fMRI data acquired during the control task

A moral dilemma decision task was performed by all subjects to localize regions involved in moral processing. The moral dilemma decision task was analyzed with a general linear model approach using the SPM12 software (www.fil.ion.ucl.ac.uk/spm). To distinguish between moments of decision in the moral dilemma and the simple perception of the presentation, we created a temporal model of the occurrence of decision moments during the experiment. The decision regressor included time points from the revelation of the identity of involved individuals to the moment of decision indicated by button press. The activity during these time points was compared to the activity in all other time points of the task, including telling the background story of the moral dilemma in the presentation. Regressors were convolved with canonical hemodynamic response function to account for hemodynamic lag. From the preprocessed input data (see above) low-frequency signal drifts were removed by high-pass filtering (cutoff 128 s). First, individual contrast images were generated for the main effects of the regressors, then first level analyses were subjected to second-level analyses in MATLAB using one-sample *t*-test to test which brain areas showed significant activations in decision vs. no decision moments in a one-sample *t*-test over subjects. Statistical threshold was set at *p* < 0.05 (cluster-corrected using the threshold free cluster enhancement approach implemented by FSL randomize with 5000 permutations).

### Recording of eye-movements

Eye movements were recorded during fMRI scanning from all subjects with an EyeLink 1000 eye tracker (SR Research, Mississauga, Ontario, Canada; sampling rate 1000 Hz, spatial accuracy better than 0.5°, with a 0.01° resolution in the pupil-tracking mode). Due to technical problems, 4 subjects had to be excluded from the final data analysis (with the rejection criteria of blinks maximum 10% of the duration of the scan and majority of blinks and saccades less than 1 second in duration). In addition, a part of recordings from some additional subjects had to de discarded due to the same criteria mentioned above, resulting in 61 recorded files with sufficient quality, with 35 files remaining in the genetic condition and 26 remaining files for the non-genetic condition. Prior to the experiment the eye tracker was calibrated once with a nine-point calibration.

Saccade detection was performed using a velocity threshold of 30°/s and an acceleration threshold of 4000°/s2. Because the experiment was relatively long and no intermediate drift correction was performed, we retrospectively corrected the mean effect of the drift. We first calculated the mean of all fixation locations over the entire experiment for each subject, and then rigidly shifted the fixation distributions so that the mean fixation location coincided with the grand mean fixation location over all subjects.

### Eye-movement analysis

Subject-wise gaze fixation distributions were compared across the genetic vs. non-genetic conditions in the movie viewing task. Individual heat maps were generated by modelling each fixation as a Gaussian function using a Gaussian kernel with a standard deviation of 1degree of visual angle and a radius of 3 standard deviations. The heat maps were generated in time windows of 2 seconds corresponding to the TR used in the fMRI measurements. Spatial similarities between each pair of heat maps across the eye-tracking sessions were calculated using Pearson’s product-moment correlation coefficient (inter-subject correlation of eye gaze, eyeISC^60^). In the end a similarity matrix was obtained with correlations between each pair for each of the 712 time windows.

First, the mean eISC scores over all 712 time windows were examined. These mean scores were acquired by extracting the mean of Fisher’s Z-transformed correlation scores and then transforming these mean values back to the correlation scale before the statistical analysis. The statistical significance of the group differences was analysed by contrasting pairs in which both subjects assumed a genetic relationship with pairs in which both subjects assumed the younger sister to be adopted. Non-parametric permutation tests with a total of 100000 permutations were used to avoid making assumptions about the data distribution. In this procedure the data were mixed randomly to change groupings and differences in the resulting new randomised groups were used to form an estimated distribution of the data. A comparison of how many of the permuted random partitions into groups build a more extreme group mean difference that the one observed with the original grouping yielded the final p-values.

### Behavioral Measurements and Self-reports

#### Valence and Arousal measurements

The subjects self-reported emotions they had experienced during movie viewing. This was carried out after the fMRI experiment by viewing the movie again (Full procedures have been described in an earlier publication^61^). Two aspects of emotional experience were rated: emotional valence (positive-negative scale) and arousal which were acquired on separate runs. While watching the movie in the middle of the screen, the subjects could move a small cursor on the right side of the screen up and down on a scale using the computer mouse to report their current state of valence or arousal using a web tool https://version.aalto.fi/gitlab/eglerean/dynamicannotations ^60^. The self-ratings were collected at 5 Hz sampling rate.

#### Behavioral questionnaires

The subjects were asked after the first fMRI session five short freeform questions about their perception of the movie, specifically about how easy it was to take one or the other perspective, and whether they would have donated their kidney if in place of the movie protagonist. After the second fMRI session all subjects were debriefed by showing them the ending of the original movie, where it is revealed that the sick sister had wished for the healthy sister to refuse donating her kidney. Afterwards they were asked if seeing the real ending changed their opinion on the roles of the two movie protagonists.

As an additional self-report measure, the subjects’ disposition for catching emotions from others was assessed with two emotional empathy questionnaires: Hatfield’s Emotional Contagion Scale^62^ and the BIS/BAS scale^63^. Every subject also filled in a questionnaire quantifying their social network^2^, including their emotional closeness to their sister and best friend. The names of the sister and best friend were obtained from this questionnaire for the moral dilemma task.

### Analysis of behavioral measurements

#### Valence and arousal measurements

To test whether dynamic valence and arousal were different between the genetic and non-genetic condition, we first computed inter-subject similarity matrices using valence and arousal rating time-series. These were compared against a similarity matrix for the experimental conditions of the viewing preceding the valence/arousal rating, i.e. the model tests for the case where individuals are more similar within the same condition (genetic or non-genetic), but dissimilar between conditions. Tests were performed using Mantel test with 5000 permutations. We also performed a test to see if subjects who were rating arousal and valence for the genetic condition had a stronger group similarity than subjects who rated arousal and valence for the non-genetic condition. Tests were performed using permutation based t-tests. As dynamic ratings can also be different in specific time points, we also performed a permutation-based t-test on valence and arousal values at each time point corrected for multiple comparisons across time.

#### Heart rate and breathing rate analysis

Differences between experimental conditions were computed in the same way as in the ISC analysis: Correlation values were first transformed into z-scores with the Fisher Z’s transform and then a permutation based approach was used to compute t-values and corresponding p-values^52^. Correction for the multiple comparisons was performed with Benjamini-Hochberg false discovery rate (BH-FDR) correction at a q < 0.05, corresponding to a t-value threshold of 2.133.

### Behavioral measurements with a new group of subjects

Subsequent to the fMRI experiments a new group of 30 subjects (all female, and having a sister, 18–33 years, mean age 25.5 years, right handed) were recruited for two further behavioral measurements. The subjects first performed an implicit association test (IAT). The IAT measures attitudes and beliefs that might not be consciously self-recognized by the subject or attitudes that the subjects are unwilling to report. By asking the subjects to sort, as quickly as possible, positively and negatively connoted words into categories, the IAT can measure the reaction times of the association process between the categories and the evaluations (e.g., good, bad). It has been shown in previous studies that making a response is easier and thus faster if the category is matching the implicit evaluation the subject bears in mind^23^. In this study the two categories were “genetic sister” (sisko) and “adopted sister” (adoptiosisko). The two categories were paired in different randomized runs with positive or negative words, thus the experiment comprised separate runs asking the subjects to either match the positive words with the category “genetic sister” and negative words with the category “adopted sister” or vice versa to match positive words with the category “adopted sister” and negative words with the category “genetic sister”. The order in which the runs are presented counter-balanced across subjects and categories switched their localization on the screen in different runs to be on the left or right side of the screen to the same extent. Subjects were asked to press a key with either the right or the left hand and thus assign the evaluation word to one category on either the left or right hand side of the computer screen. With the experiment going on, the number of trials in this part of the IAT is increased in order to minimize the effects of practice. The IAT score is based on how long it takes a person to sort the words with the condition associating positive words and genetic (and negative and adopted) in contrast to negative words and genetic (and positive words and adopted). If an implicit preference exist for one of the categories subjects would be faster to match positive words to that category relative to the reverse. Data were analysed using Matlab. Similarity between subjects’ scores were examined TOST testing^34^. As a second task, reaction times for the moral decision task were measured with the same group of subjects that underwent the IAT. As a difference to the decision task performed during fMRI scanning the order of the decisions was randomized (with easy decision including only strangers and difficult decisions including the sister on one side and the friend on the other). Reaction times were measured as the time between the onset of the slide revealing the identity of the involved individuals and the button press of the subject that related her decision.

### Data availability

The data that support the findings of this study are available on request from the corresponding author MBT. The data are not publicly available due to a prohibition by the Finnish law: Juridical restrictions set by the Finnish law prevent public access to the collected data, be it anonymized or non-anonymized, when data are recorded from human individuals. As the consent given by the subjects only applies to the specific study reported in our manuscript, no portion of the data collected could be used or released for use by third parties.
